# Trophoblast differentiation, invasion and hormone secretion in a three-dimensional in vitro implantation model with rhesus monkey embryos

**DOI:** 10.1186/s12958-018-0340-3

**Published:** 2018-03-16

**Authors:** T. Arthur Chang, Gennadiy I. Bondarenko, Behzad Gerami-Naini, Jessica G. Drenzek, Maureen Durning, Mark A. Garthwaite, Jenna Kropp Schmidt, Thaddeus G. Golos

**Affiliations:** 1 Wisconsin National Primate Research Center, University of Wisconsin-Madison, 1223 Capitol Court, Madison, Wisconsin 53715-1299 USA; 2 Department of Comparative Biosciences, School of Veterinary Medicine, University of Wisconsin-Madison, Madison, WI USA; 3 Department of Obstetrics and Gynecology, School of Medicine and Public Health, University of Wisconsin-Madison, Madison, WI USA; 4 Present address: Department of Obstetrics and Gynecology, University of Texas Health Science Center, San Antonio, TX USA; 5 Present address: Covance Laboratories, Madison, WI USA; 6 Present address: School of Dental Medicine, Tufts University, Boston, MA USA; 7 Present address: Illumina-Madison, Madison, WI USA

**Keywords:** Embryo, Implantation, Trophoblast, Non-human primate

## Abstract

**Background:**

The initiation of primate embryo invasion into the endometrium and the formation of the placenta from trophoblasts, fetal mesenchyme, and vascular components are essential for the establishment of a successful pregnancy. The mechanisms which direct morphogenesis of the chorionic villi, and the interactions between trophectoderm-derived trophoblasts and the fetal mesenchyme to direct these processes during placentation are not well understood due to a dearth of systems to examine and manipulate real-time primate implantation. Here we describe an in vitro three-dimensional (3-D) model to study implantation which utilized IVF-generated rhesus monkey embryos cultured in a Matrigel explant system.

**Methods:**

Blastocyst stage embryos were embedded in a 3-D microenvironment of a Matrigel carrier and co-cultured with a feeder layer of cells generating conditioned medium. Throughout the course of embryo co-culture embryo growth and secretions were monitored. Embedded embryos were then sectioned and stained for markers of trophoblast function and differentiation.

**Results:**

Signs of implantation were observed including enlargement of the embryo mass, and invasion and proliferation of trophoblast outgrowths. Expression of chorionic gonadotropin defined by immunohistochemical staining, and secretion of chorionic gonadotropin and progesterone coincident with the appearance of trophoblast outgrowths, supported the conclusion that a trophoblast cell lineage formed from implanted embryos. Positive staining for selected markers including Ki67, MHC class I, NeuN, CD31, vonWillebrand Factor and Vimentin, suggest growth and differentiation of the embryo following embedding.

**Conclusions:**

This 3-D in vitro system will facilitate further study of primate embryo biology, with potential to provide a platform for study of genes related to implantation defects and trophoblast differentiation.

**Electronic supplementary material:**

The online version of this article (10.1186/s12958-018-0340-3) contains supplementary material, which is available to authorized users.

## Background

Implantation is a crucial developmental event for establishing a pregnancy. The central events to implantation are the apposition of the blastocyst to the uterine epithelium, adhesion of the embryo to the uterus, followed by the penetration and invasion of trophoblast cells into the uterine stroma [1]. Abnormal implantation of an embryo is an underlying cause of infertility and has implications in development of placenta-associated pathologies including preclampsia and intrauterine growth restriction. The understanding of the mechanisms driving the key events of human implantation, particularly those that lead to placental pathologies, remains unclear. Given the ethical constraints in assessing human implantation in vivo, animal models and in vitro platforms are needed to study mechanisms underlying the establishment of pregnancy.

Current in vitro models of human implantation mimic interactions between the embryo and endometrium, as reviewed by Hohn and Denker [2] and Weimar et al. [3], however, these models focus on a specific stage of implantation rather than fully recapitulating the entire process. Human in vitro implantation models are comprised of either a human embryo or an embryo surrogate, such as trophoblast spheroids, positioned above either a layer of endometrial stromal and/or epithelial cells, or alternatively, a synthetic basement membrane [3–6]. Although these models garnered valuable knowledge on trophoblast attachment [6–8], migration [9], invasion [4] and molecular interactions at the interface [9, 10], there are also limitations to these models. There are few studies utilizing human embryos due to the ethical constraints surrounding their use. As a substitute, embryo surrogates, such as trophoblast spheroids, derived from immortal cell lines have been utilized to evaluate attachment, invasion and migration [6, 7, 11, 12]. These models are also limited to studying a specific event during implantation and lack a complete representation of all cell types present at the maternal-fetal interface, including immune cells, thus further development of in vitro implantation models that better simulate in vivo implantation are needed.

Animal models are particularly effective for studying implantation both in vivo and in vitro [1, 13], and bypass the challenges posed by human models. However, the degree of penetration and invasion of the embryo into the uterus varies greatly across species [1]. When considering a rodent model, mouse models are useful for assessing genes and gene networks involved in implantation, however, placental organization and the invasion of trophoblasts into the uterine epithelium is relatively shallow in comparison to the human. The placentation and endovascular invasion of the human is more closely paralleled in non-human primates including the great apes, baboons and macaques [13, 14]. Despite the high cost of maintaining primate colonies in comparison to rodents, the use of assisted reproductive technologies can be implemented to increase the number of embryos produced by a single animal. In vitro studies of blastocyst formation in the marmoset [15–17], baboon [18], rhesus monkey [19–21] and human [22] demonstrated the feasibility of embryonic development to the blastocyst stage in vitro following IVF. Such studies also showed that in vitro cultured preimplantation primate embryos could produce chorionic gonadotrophin (CG) [23–25] and syncytiotrophoblast-like cells when cultured beyond the time of normal implantation [18, 24, 26]. This supports the possibility that extended primate embryo culture, under appropriate conditions, could provide a paradigm for development of new approaches to study mechanisms underlying primate peri-implantation development.

A central feature to developing an in vitro model of embryo implantation is creating a microenvironment that supports development similar to that observed in vivo. Three-dimensional culture systems may create a better microenvironment, including an extracellular matrix (ECM) that supports adhesion and regulates cellular proliferation and differentiation [27, 28]. When considering a three-dimensional (3-D) in vivo implantation scenario [29–32], extrapolating results from 2-D models to the physiological situation are likely to be simplistic. In a previous study by Gerami-Naini et al. [33], significant differences between embryoid bodies derived from human embryonic stem cells cultured in 2-D and 3-D systems were observed in the maintenance of a differential trophoblast phenotype, as defined by CG and progesterone secretion. Moreover, that study reported substantial outgrowth of trophoblasts into Matrigel (MG), coincident with elevated CG secretion. Based on this study, we have adopted a 3-D model to study primate embryo development in vitro. The current study presented demonstrates that an in vitro 3-D environment can promote non-human primate embryo development that manifests characteristics of in vivo implantation and pregnancy initiation. Overall, the 3-D implantation model builds on knowledge obtained from traditional 2-D culture environments, and provides a platform to study effects and impact of various growth factors or experimental conditions thought to be critical for the local support of implantation.

## Methods

### In vitro production of rhesus monkey embryos

Rhesus monkeys (*Macaca mulatta*) were from the colony maintained at the Wisconsin National Primate Research Center. All procedures were performed in accordance with the NIH Guide for the Care and Use of Laboratory Animals and under the approval of the University of Wisconsin Graduate School Animal Care and Use Committee. In vitro production of rhesus macaque embryos was carried out as previously reported by others [34–40] and are described here in brief. Rhesus macaque (*Macaca mulatta*) oocyte donors were hyperstimulated by recombinant human follicle stimulation hormone (FSH; Organon, Oss, Netherlands) 30 IU per injection twice daily for 7 days, followed by a single injection of 1000 IU human chorionic gonadotropin (hCG; Serono S.A., Geneva, Switzerland) on day 8. Cumulus-oocyte complexes were retrieved by laparoscopic follicular aspiration 27–30 h after hCG administration and placed in TL-Hepes medium containing 0.3% bovine serum albumin at 37 °C. Aspirated contents were filtered through an EmCon filter (Veterinary Concepts, Spring Valley, WI) and immediately washed with TL-Hepes media containing hyaluronidase (1 mg/ml). Oocytes were stripped of residual cumulus cells by mechanical pipetting, and placed 10 per 50 μl drop of CMRL-1066 media, supplemented with 20% fetal bovine serum, 0.5 mM sodium pyruvate, 2 mM l-alanyl-l-glutamine, until fertilization. Prior to fertilization, semen was collected from male rhesus monkey donors, washed and capacitated in TL-based capacitation medium (containing 0.1% sodium Pyruvate, 0.01% Polyvinyl alcohol) covered by mineral oil prior to fertilization. Matured oocytes and capacitated sperm were moved to TL-based IVF medium containing 0.5% sodium pyruvate, 1.0% glutamine and 0.01% PVA for fertilization. Fertilized embryos were cultured to the 8-cell stage at 37 °C in 5% CO_2_, 5% O_2_, and 90% N_2_ in chemically defined, protein-free hamster embryo culture medium-9 (HECM-9) media [38, 41, 42] supplemented with amino acids. Embryos at the 8-cell stage were selected and transferred to fresh plates of HECM-9 supplemented with amino acids and 5% fetal bovine serum (FBS; HyClone, Logan, UT) with medium changed every other day. Embryos were cultured for a maximum of 9 days at which point blastocyst stage embryos were embedded into a Matrigel (MG) raft for extended culture.

### Preparation of Matrigel rafts and extended embryo culture

As previously described by Gerami-Naini et al. [33], dome-shaped, growth factor-reduced MG (BD-Biosciences, Bedford, MA) carriers were formed by initially pipetting 50–60 μl MG onto sterile glass cover slides, followed by sequential pipetting of additional 30 μl volumes of MG, within 5 min intervals to allow for the previous layer to solidify. Embryos at the morula and blastocyst stages (6 to 8 days post insemination) were transferred by a micropipettor and embedded into the MG raft. A feeder layer of Buffalo Liver Rat (BRL) cells were plated onto the bottom of 35 mm 6-well dishes and cultured for 1 day prior to receiving embryo rafts to establish a conditioned culture environment. Each MG raft containing 1 embryo was placed into one 35 mm well containing BRL-conditioned media and the feeder layer cells (Additional file 1). Morphological assessment of embryo development was performed included the time of hatching, initiation of MG invasion and outgrowth, sizes and shape of outgrowth and changes of outgrowth proliferation, and degradation of outgrowths. Photomicrographic images were captured using a Leica DMIRB microscope and MagnaFire software. Media were collected every 3 days for hormone assays, and the rafts were transferred to fresh feeder dishes at the same time. Collected media were briefly centrifuged and the supernatants were stored at − 20°C until hormone assays were performed. While most embryos were terminated by the end of the third week of culture, some embryos were maintained for up to 45 days in MG.

### Hormone assays

Levels of CG in the conditioned embryo media samples were determined by Leydig cell bioassay as previously described [43]. The minimum detectable level was 10 pg of hCG/tube, and the intraassay and interassay coefficients of variation were 5.34% and 9.77% respectively. Progesterone secretion was determined by enzyme immunoassay [44–46]. All hormone determinations were made on duplicate samples.

### Immunohistochemistry (IHC)

For histological and IHC analyses, MG-embedded embryos were removed from cover slips at various time points with minimal amount of MG carry over, fixed for 2 h in 2% paraformaldehyde, washed 3 times in PBS, then covered by 1% agarose warmed to 48°C. Agarose containing the MG-embedded embryo was rapidly cooled by placement on ice, dehydrated and embedded in paraffin. Trimmed blocks containing an embryo were sectioned at 5 μm for IHC staining procedures. For CD31 and MHC class I IHC, 5 μm paraffin sections were treated with proteinase K as previously described [33, 47]; for all other IHC experiments, sections were boiled in a microwave oven using sodium citrate buffer (pH 6.0) [33, 47]. Antibodies are listed in Additional file 2. Negative controls were isotype-matched rabbit or mouse IgG (Sigma), at the same concentration as the specific primary antibodies. Endogenous peroxidase activity was quenched using 5% hydrogen peroxide in methanol for 20 min, and sections were blocked by incubation for 30 min with 20% horse serum in 4% TBS/Triton X-100. Positive immunostaining was visualized using an ABC Peroxidase Kit (Vector Labs, Burlingame, CA) and freshly prepared Nova Red substrate (Vector Labs). Sections were counterstained with hematoxylin, mounted in organic mount Cytoseal XYL (both from Richard-Allan Scientific, Kalamazoo, MI), and analyzed by light microscopy.

## Results

### Morphological observations of embryo development

**Fig. 1:**
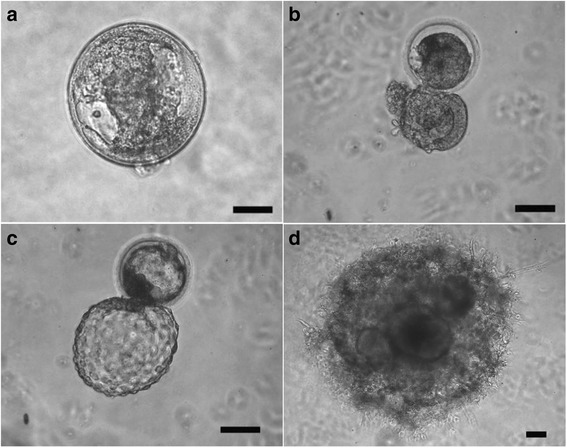
Growth of Rhesus Embryos in Matrigel Rafts. Embryos that failed to hatch (**a**) or that did not completely hatch (**b**), failed to develop and degenerated. Embryos that completed hatching (**c**) continued to develop (**d**), where within 15 days post embedding trophoblast protrusions and inner cavities formed. Scale bar = 100 μm

**Fig. 2:**
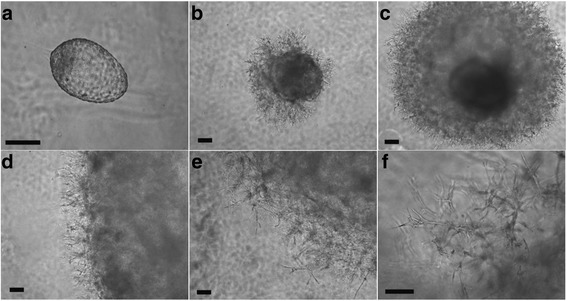
Development of rhesus blastocysts embedded in Matrigel: (**a**) day 0 post embedding in Matrigel; (**b**) day 6: outgrowths and cystic structure shown; (**c**) day 14: extensive branch-like outgrowths extended from dense extraembryonic mass; (**d**) day 19; (**e**) day 26; (**f**) day 38. Over the course of development post-embedding, trophoblastic protrusions invaded into the Matrigel environment. Scale bar = 100 μm

Embryonic escape from the zona pellucida, or hatching, is a critical step required for implantation and further differentiation and development. In initial experiments, we determined whether embryos transferred and embedded into a “raft”, prepared from a 100 μl drop of MG, at the zona-enclosed blastocyst stage were capable of hatching from the zona pellucida. Embryos were morphologically evaluated following embedding in the raft. Embryos that successfully hatched routinely displayed growth of the embryonic mass and outgrowth of cells into the MG, whereas those that did not complete hatching failed to further develop. Representative images of the development of 16 rhesus embryos embedded into MG are shown in Figs. 1, 2 and 4. In pilot experiments, embryos transferred into MG at the zona-enclosed morula or earlier stages did not hatch within the MG; hence no further development was observed. This is not unexpected since embryo implantation does not occur at the morula stage of development. Morula stage embryos that had the zona pellucida removed in vitro and were then embedded into MG likewise did not progress into blastocyst and develop outgrowths (not shown), while blastocysts that underwent assisted hatching continued to develop. Zona-intact blastocysts that received assisted hatching prior to implantation into the MG rafts, developed outgrowth protrusions in the MG, similar to their spontaneously hatched counterparts. Therefore, assisted hatching was applied to all embryos prior to implantation into the MG rafts in subsequent experiments to provide a consistent experimental paradigm.

The initiation of invasion by embryo-derived cells was heralded as a visible spherical enlargement of the embryo mass and a thickened layer of trophectoderm observed after zona escape was completed (Fig. 2b,c). Within the first week post-embedding, the diameter of the embryo mass increased by two to three-fold greater than the hatching stage. This spherical enlargement, accompanied by a thicker layer of cells, putatively derived from the trophectoderm of the blastocyst, was a strong indicator of future development of outgrowths. In addition, the formation of “cystic” structures inside the embryo or appearing at the surface of the embryonic trophectoderm layer was noted (Additional file 3A). Formation of the “cystic” appearing structures began by the end of the first week post-embedding, continued to enlarge during the early stages of protrusion growth, and was retained while further trophoblast outgrowth continued (Fig. 2c; Additional file 3B). The specific identity of cells involved in formation of these cystic structures remains to be defined.

Embryos which successfully expanded typically progressed to show varied levels of development of extraembryonic structures with significant outgrowths of a variety of morphologies, including elongated branches, as well as dense shell-like cellular structures (Fig. 2c, d). Approximately a week after embedding into the MG, 30% of embryos typically began to exhibit these projections (Figs. 1 and 2), which were similar in appearance to trophoblastic outgrowths observed with embryoid bodies derived from human embryonic stem cells [33]. Once initiated, the protrusions typically continue to extend and elongate throughout the culture period, with the length of the embryonic outgrowths extending to 7 mm, although heterogeneity was seen among embryos (Additional file 3). Overall, 15 of 16 embryos with trophoblastic protrusions grew through day 18, 2 were allowed to continue to day 21, and 2 progressed to day 45 at which point embryos were terminated for analysis.

### Hormone secretion by cultured embryos into culture media

**Fig. 3:**
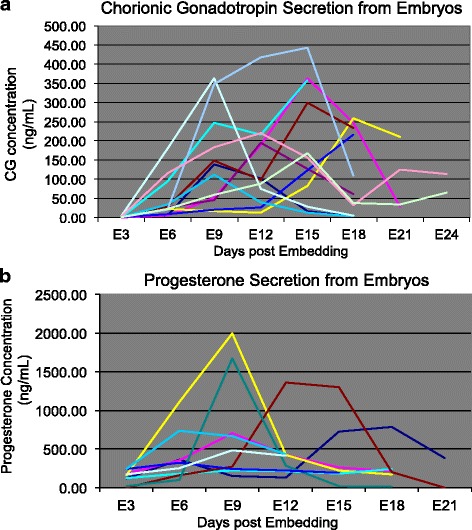
Hormone secretion from embryos embedded in Matrigel and co-cultured with BRL cells: (**a**) CG, *n* = 12 and (**b**) progesterone secretion, *n* = 9. Each individual colored line represents the secretion profile from an individual embryo, where the same color between (**a**) and (**b**) represent the same embryo

**Fig. 4:**
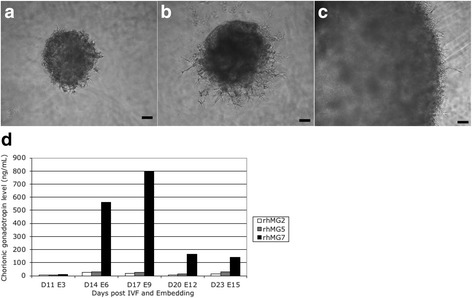
Comparison of chorionic gonadotropin secretion in embryos derived from the same oocyte donor with less extensive development (**a**, **b**) or advanced development (**c**). Chorionic gonadotropin secretion profiles, illustrated in panel (**d**), from those three embryos showed higher CG secretion coincident with more advanced development of the embryo and trophoblastic shell. Scale bar = 100 μm

Culture media were evaluated for secretion by the embryo of both CG and progesterone, which are markers of primate trophoblast differentiation. As illustrated in Fig. 3, secretion of CG and progesterone was detectable in culture media from embryos within the first week post-embedding. Elevated levels of secreted CG were sometimes observed with more developmentally advanced embryos (Fig. 4). We evaluated the secretion of CG in association with embryo growth in Matrigel. Additional file 4 illustrates that there was a trend for peak CG secretion to be correlated with embryo size, however this trend was not statistically significant (*P* = 0.09). Although trophoblastic outgrowths continued to expand throughout culture, levels of CG and progesterone approached basal levels by the end of the third week of culture (Fig. 3).

### Histological evaluation of ECM-cultured rhesus embryos

**Fig. 5:**
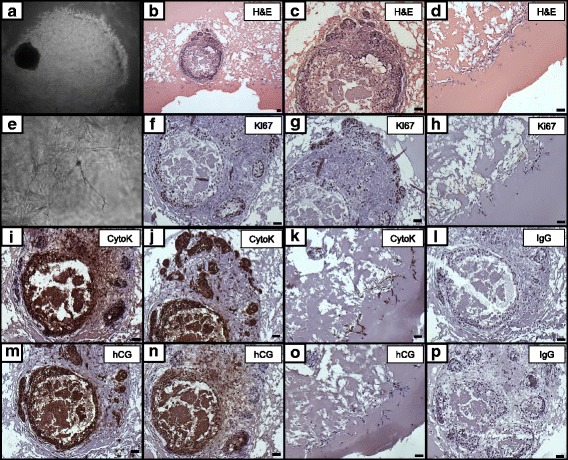
Histological and immunohistochemical images of paraffin sections of embedded in vitro developed rhesus embryos: (**a**) bright field; (**b**-**d**) H&E; (**e**) bright field image of trophoblastic outgrowths; (**f**-**h**) Ki67; (**i**-**k**) cytokeratin (CytoK); (**l**) IgG negative control for cytokeratin; (**m**-**o**) CG; and (**p**) IgG negative control for CG. Results representative of 2 embryos. Scale bar = 500 μm

To distinguish cellular structures across various time points of culture, embryos were embedded in paraffin, sectioned and evaluated by hematoxylin and eosin (H&E) and IHC. Embryos terminated around day 45 post-embedding demonstrated extensive outgrowth development of invasive trophoblasts, as indicated by the bright “halo” around the embryo mass, represented in the bright field images of Fig. 5. Sections stained with H&E revealed extensive digestion of the MG environment presumably by the outgrowths expanding and migrating from the surface of the embryo (Fig. 5c). The leading edge of the outgrowths were seen to be individual cells that were migrating away from the embryo proper (Fig. 5b, c), leaving a substantially degraded MG in the vicinity of the embryo, whereas the MG distant from the embryo had a homogeneous, undisturbed, eosinophilic appearance by H&E staining.

Proliferation of embryo-derived cells was evaluated by IHC for Ki67. As the embryo progressed in development, Ki67 was extensively noted primarily in cells at the cytokeratin-positive periphery of the expanded embryonic structure (Fig. 5h), as well as in the migrating cells which had invaded into the MG, and were distant from the embryo proper (Fig. 5f-g).

Characterization of embryonic and extraembryonic differentiation was assessed by IHC of embryos terminated early and late in culture. IHC with an antibody recognizing cytokeratin 7/8 demonstrated multiple areas of epithelial differentiation, including potential trophoblast formation. Consistently, an outer layer of cytokeratin-positive cells was observed at the border of the expanded embryo (Fig. 5i, j). We also observed positive cytokeratin staining of the migrating individual cells at the outer edge of the overall outgrowth shell (Fig. 5i, j), identifying the protrusions as trophoblast cells mimicking endometrial invasion.

Immunostaining for CG was performed to determine whether CG was expressed within the embryos. Immunostaining with antibodies against CG demonstrated that the primary localization of CG-β subunit expression was in the cytokeratin-positive layer constituting the outer surface of the embryo. As the embryo progressed in development, CG staining was positive in the embryonic structures proper as well as extraembryonic protrusions (Fig. 5m-o), including internal localization of CG within the central cavity of the embryo, which was clearly specific in comparison with staining of nonspecific control IgG (Fig. 5p) or other antibodies (Fig. 5).

**Fig. 6:**
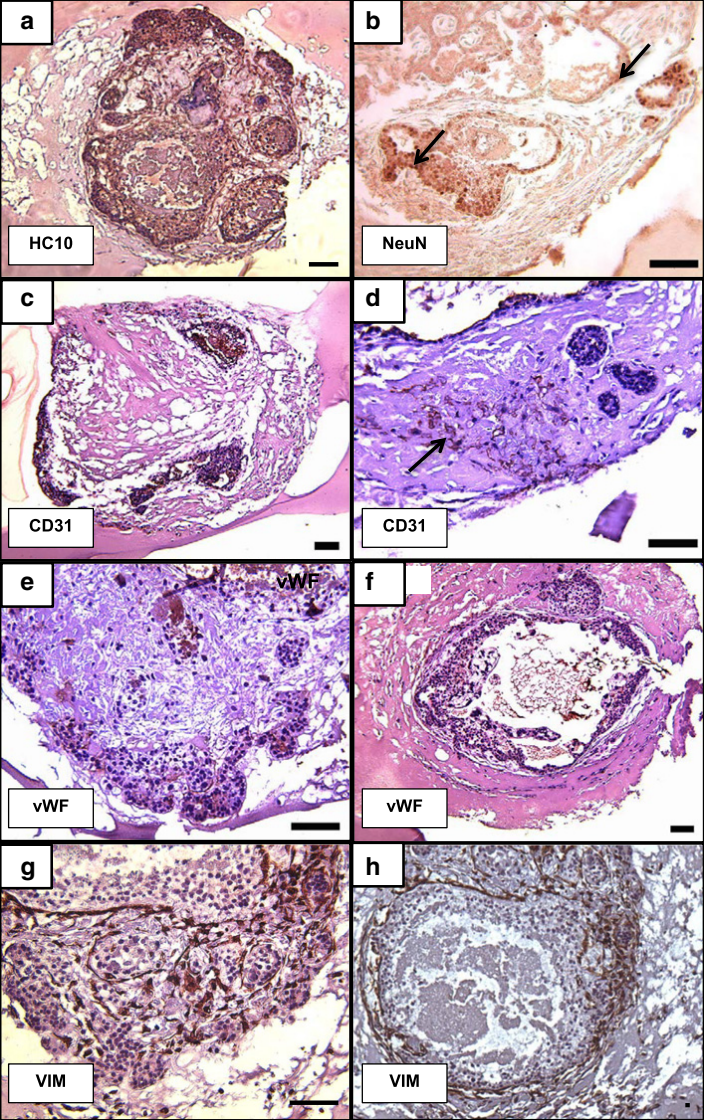
Immunohistochemical staining for selected markers in Matrigel-embedded embryos: (**a**) HC10, (**b**) neuronal marker NeuN; (**c**-**d**) CD31, an endothelial and monkey extravillous trophoblast marker; (**e**-**f**) von Willebrand Factor (vWF), an endothelial marker (arrow); (**g**-**h**) Vimentin (VIM), a marker of mesenchymal cells. Results representative of 2 embryos. Scale bar = 100 μm

To better characterize the cells which comprise the embryo at later stages of development, IHC was performed for several selected markers indicative of differentiation. Evaluation of MHC class I expression by the HC10 antibody identified MHC class I heavy chains widely expressed throughout the embryo (Fig. 6a). Neuroectoderm differentiation was also evaluated by staining with NeuN antibodies, which showed positive staining on embryonic structures (Fig. 6b). CD 31, an endothelial cell marker, and in the rhesus, a marker for differentiated trophoblasts [48], was also used to determine these cell lineages in embryos growing in MG. Staining for CD31 was apparent in the areas surrounding the villous embryonic structure, as well as part of the trophoblast shell (Fig. 6c, d). Positive staining for the endothelial marker, von Willebrand Factor (vWF), was also observed in the outer villous area and cells lining the embryonic structure (Fig. 6e, f), suggesting endothelial cell differentiation. Staining with an anti-vimentin antibody, a marker of mesenchymal cells, showed positive expression in cords of fibroblast-like cells (Fig. 6g, h). Collectively, these results demonstrate that the embryonic cells undergo differentiation, however, a more rigorous characterization is warranted in future studies.

## Discussion

Here we describe an experimental platform for long term in vitro rhesus embryo culture using a novel 3-D Matrigel scaffold. Similar to in vivo implantation, extensive trophoblast migration into the extracellular, MG environment was observed. The extensive degradation of the MG at the interface with the embryo, and the migration of these cells suggests the secretion of proteolytic enzymes to facilitate migration, however this remains to be demonstrated formally. Trophoblast differentiation from embryos maintained in MG explants was demonstrated by the secretion of CG and progesterone into the culture medium, and CG expression was confirmed by IHC analyses. Differentiation within the embryo and the extraembryonic structures, as well as sustained proliferation within these cultured embryos, demonstrates that this 3-D in vitro system provides a unique paradigm to model early events in primate implantation, the initiation of pregnancy, and the determination of trophoblast lineage specification in primate development.

Ethical limitations surrounding the study of human implantation in vivo has driven the need to establish in vitro models of implantation to better understand the molecular mechanisms of implantation. Human in vitro models of implantation include embryos or embryo surrogates, such as a trophoblast spheroid, cultured on a specified ECM, layer(s) of endometrial cells, or placed into a transwell setup or bioreactor [2, 3, 30, 49]. There are limitations to these models, including the ethical concern surrounding the use of human embryos, the use of immortalized trophoblasts and/or endometrial epithelial cell lines compared to primary cells, and the technical challenge of reproducibly isolating populations of primary endometrial epithelial and stromal cells.

To overcome these challenges, our model rather relies on a non-human primate embryo embedded within a synthetic ECM, MG, to define the feasibility of studying embryonic development during primate implantation. Our study illustrated that the embryo itself can interact with its surrounding environment to support embryonic growth, and initiate trophoblast proliferation and differentiation in the absence of endometrial cells. Enders et al. [50] previously reported that 2-dimensional culture of rhesus blastocysts on MG alone resulted in inappropriate development and limited trophectoderm outgrowth. In our 3-D environment, branches and protrusions from the embedded rhesus embryos that rapidly developed in MG as similarly described in human embryoid bodies [33], non-human primate embryos [24], and human embryos [4]. Interaction between the embryo and the MG ECM was observed based on the development of trophoblast columns extending from the embryo proper accompanied by apparent proteolytic degradation of the ECM from the invading trophoblasts. The adhesion of the embryo-derived structures and cells to the MG, and the involvement of specific proteins are beyond the scope of this study, but are important areas for future investigation.

During placenta formation in vivo, the highly proliferative and invasive extravillous trophoblast (EVT) structures migrate and invade the uterus and its vasculature. Previous studies have demonstrated that migration and invasion of human EVTs are regulated by growth factors, their binding proteins, ECM components and adhesion molecules, in autocrine and paracrine manners at the maternal-fetal interface in human pregnancy [51–53]. In the present study, embryonic cell migration was concomitant to degradation of the MG in the area surrounding protrusions was observed. These events were similarly observed for human in vitro first trimester trophoblasts, where upon attachment trophoblasts subsequently migrated through a matrix-substrate [54, 55]. Degradation of the ECM is likely a result of secretion of matrix metalloproteinases (MMPs) from the trophoblast cells as similarly observed in vivo during implantation. A critical process to establishment of pregnancy is trophoblast cell invasion into the uterus and modification of maternal spiral arteries, and one of the major factors contributing to the process is the secretion of MMPs [54, 56].

Extraembryonic cellular differentiation in our model was evident by the secretion of CG, a hallmark of trophoblast differentiation to syncytiotrophoblasts [57]. Consistently elevated CG and progesterone secretion, coincident with the presence of outgrowths confirmed trophoblast differentiation. The presence of trophectodermal growth was absolutely required for the secretion of CG: in occasional embryos which failed to initiate trophectodermal proliferation and expansion, CG was essentially never detectable. Interestingly, CG secretion was sustained for several weeks in embryos, similar to the in vivo hormonal secretion in the rhesus monkey [35]. While the secretion of CG in rhesus monkey embryos has been previously described [40, 58], the present study is the first to report immunohistochemical localization of CG in a 3-D in vitro cultured rhesus embryo model.

Proliferation and differentiation of the embryonic and extraembryonic cells was further confirmed by immunohistochemistry for various selected markers. Cellular proliferation was evidenced by positive staining of Ki67 throughout the interior of the embryonic structures with a greater proportion of positive at the periphery of outgrowth, thus demonstrating continued growth throughout the duration of culture. At the periphery of outgrowth positive Ki67 cells were co-localized with cytokeratin positive cells. Cytokeratin 7 is a well-known marker for the characterization and identification of differentiated trophoblasts in placental cell isolates [59–61]. Conversely, vimentin-positive cells were observed internally in the embryonic structures, complementary to cytokeratin staining. Vimentin is a non-trophoblastic, mesenchymal marker and is expressed in fibroblasts and endothelial cells [62].

Our previous study identified a non-human primate MHC class I gene, Mamu-AG, which has similar characteristics to the human MHC class I gene, HLA-G [63]. The expression of Mamu-AG is restricted to the placenta, although a soluble form is also expressed in the testis of rhesus macaques [64, 65]. Temporal expression pattern of Mamu-AG has been observed at implantation sites of rhesus macaque placentas, where at days 10–14 of pregnancy it is expressed predominantly in cytotrophoblasts and as development progresses there is an accumulation of the glycoprotein at the interface between the decidua and trophoblast by day 36 of pregnancy [47]. Although the paraffin embedding of the embryos precluded specific staining in the present study, the HC10 antibody revealed that MHC Class I molecules are expressed throughout the embryonic structure, including cells at the leading edge, consistent with a differentiated trophoblast phenotype.

Immunohistochemistry confirmed the presence of cellular differentiation by positive staining for other selected markers including CD31, vWF, and NeuN. CD31 and vWF staining on the outer areas of the embryonic structures indicating possible development of endothelial cells as well as differentiated trophoblasts in the post-implantation rhesus macaque embryo. NeuN positive staining on the embryonic structures suggests the formation of neuroectoderm cell lineages in the 3-D model cultured embryo. The presence of cells positive for these markers confirms differentiation, however, further characterization of cellular differentiation representative of the respective germ layers is needed in future studies.

## Conclusions

Overall, the results reported here establish a platform for assessing primate implantation in vitro. This non-human primate 3-D model, because of its relative simplicity, isolates the embryonic component during implantation allowing for investigators to assess the impact of additional variables added to the MG, such as growth factors or maternal immune or uterine cells. The 3-D implantation model could be used in an experimental infection paradigm to evaluate the impact of pathogens on trophoblast differentiation and function. Altogether, our 3-D non-human primate model could serve as valuable platform to for study of the mechanisms underpinning human implantation.

## Additional files

Additional file 1: Schematic illustration of rhesus macaque embryo culture in the Matrigel-feeder cell 3-D in vitro implantation system. The Matrigel carrier was gelled onto a coverslip then placed into a 35 mm well containing feeder layer of Buffalo Rat Liver cells, followed by injection of one rhesus macaque blastocyst stage embryo into each Matrigel carrier. (PPTX 41 kb)

Additional file 2: Antibodies used for immunohistochemical staining on rhesus macaque embryo sections. (DOCX 72 kb)

Additional file 3: Branching structure of the trophoblastic protrusion derived from embryo embedded in Matrigel with BRL feeder cell co-culture. Scale bar = 100 μm. (PPTX 715 kb)

Additional file 4: Secretion of CG correlated with embryo size. The peak secretion of CG into culture medium in embryos presented in Fig. 3 is plotted against the maximal growth of embryos through days 18–21 of culture. The line presented was derived by best-fit linear regression analysis. Estimation of the correlation coefficient (Spearman’s nonparametric test) indicted that the correlation was not statistically significant (*P* = 0.093). (PDF 206 kb)

## Abbreviations

3-D: three-dimensional
BRL cells: Buffalo rat liver cells
CG: Chorionic gonadotropin
ECM: Extracellular matrix
EVT: Extravillous trophoblasts
HECM: Hamster embryo culture medium
IHC: Immunohistochemistry
MG: Matrigel
MMP: Matrix metalloproteases
vWF: von Willebrand Factor

## References

[1] Lee KY, DeMayo FJ (2004). Animal models of implantation. Reproduction.

[2] Hohn HP, Denker HW (2002). Experimental modulation of cell-cell adhesion, invasiveness and differentiation in trophoblast cells. Cells Tissues Organs.

[3] Weimar CH, Post Uiterweer ED, Teklenburg G, Heijnen CJ, Macklon NS (2013). In-vitro model systems for the study of human embryo-endometrium interactions. Reprod BioMed Online.

[4] Carver J, Martin K, Spyropoulou I, Barlow D, Sargent I, Mardon H (2003). An in-vitro model for stromal invasion during implantation of the human blastocyst. Hum Reprod.

[5] Bentin-Ley U, Pedersen B, Lindenberg S, Larsen JF, Hamberger L, Horn T (1994). Isolation and culture of human endometrial cells in a three-dimensional culture system. J Reprod Fertil.

[6] Wang H, Pilla F, Anderson S, Martinez-Escribano S, Herrer I, Moreno-Moya JM, Musti S, Bocca S, Oehninger S, Horcajadas JA (2012). A novel model of human implantation: 3D endometrium-like culture system to study attachment of human trophoblast (jar) cell spheroids. Mol Hum Reprod.

[7] Ho H, Singh H, Aljofan M, Nie G (2012). A high-throughput in vitro model of human embryo attachment. Fertil Steril.

[8] Bentin-Ley U, Horn T, Sjogren A, Sorensen S, Falck Larsen J, Hamberger L (2000). Ultrastructure of human blastocyst-endometrial interactions in vitro. J Reprod Fertil.

[9] Lacey H, Haigh T, Westwood M, Aplin JD (2002). Mesenchymally-derived insulin-like growth factor 1 provides a paracrine stimulus for trophoblast migration. BMC Dev Biol.

[10] Meseguer M, Aplin JD, Caballero-Campo P, O'Connor JE, Martin JC, Remohi J, Pellicer A, Simon C (2001). Human endometrial mucin MUC1 is up-regulated by progesterone and down-regulated in vitro by the human blastocyst. Biol Reprod.

[11] Holmberg JC, Haddad S, Wunsche V, Yang Y, Aldo PB, Gnainsky Y, Granot I, Dekel N, Mor G (2012). An in vitro model for the study of human implantation. Am J Reprod Immunol.

[12] Hohn HP, Linke M, Denker HW (2000). Adhesion of trophoblast to uterine epithelium as related to the state of trophoblast differentiation: in vitro studies using cell lines. Mol Reprod Dev.

[13] Carter AM (2007). Animal models of human placentation--a review. Placenta.

[14] Carter AM, Enders AC, Pijnenborg R (2015). The role of invasive trophoblast in implantation and placentation of primates. Philos Trans R Soc Lond Ser B Biol Sci.

[15] Gilchrist RB, Nayudu PL, Hodges JK (1997). Maturation, fertilization, and development of marmoset monkey oocytes in vitro. Biol Reprod.

[16] Marshall VS, Browne MA, Knowles L, Golos TG, Thomson JA (2003). Ovarian stimulation of marmoset monkeys (Callithrix jacchus) using recombinant human follicle stimulating hormone. J Med Primatol.

[17] Sasaki E, Suemizu H, Shimada A, Hanazawa K, Oiwa R, Kamioka M, Tomioka I, Sotomaru Y, Hirakawa R, Eto T (2009). Generation of transgenic non-human primates with germline transmission. Nature.

[18] Pope CE, Pope VZ, Beck LR (1982). Development of baboon preimplantation embryos to post-implantation stages in vitro. Biol Reprod.

[19] Wolf DP, Vandevoort CA, Meyer-Haas GR, Zelinski-Wooten MB, Hess DL, Baughman WL, Stouffer RL (1989). In vitro fertilization and embryo transfer in the rhesus monkey. Biol Reprod.

[20] Zhang L, Weston AM, Denniston RS, Goodeaux LL, Godke RA, Wolf DP (1994). Developmental potential of rhesus monkey embryos produced by in vitro fertilization. Biol Reprod.

[21] Bavister BD, Boatman DE, Leibfried L, Loose M, Vernon MW (1983). Fertilization and cleavage of rhesus-monkey oocytes Invitro. Biol Reprod.

[22] Niakan KK, Han J, Pedersen RA, Simon C, Pera RA (2012). Human pre-implantation embryo development. Development.

[23] Fishel SB, Edwards RG, Evans CJ (1984). Human chorionic gonadotropin secreted by preimplantation embryos cultured in vitro. Science.

[24] Lopata A, Kohlman DJ, Bowes LG, Watkins WB (1995). Culture of marmoset blastocysts on matrigel: a model of differentiation during the implantation period. Anat Rec.

[25] Lopata A, Oliva K, Stanton PG, Robertson DM (1997). Analysis of chorionic gonadotrophin secreted by cultured human blastocysts. Mol Hum Reprod.

[26] Enders AC, Boatman D, Morgan P, Bavister BD (1989). Differentiation of blastocysts derived from in vitro-fertilized rhesus monkey ova. Biol Reprod.

[27] Schindler M, Nur EKA, Ahmed I, Kamal J, Liu HY, Amor N, Ponery AS, Crockett DP, Grafe TH, Chung HY (2006). Living in three dimensions: 3D nanostructured environments for cell culture and regenerative medicine. Cell Biochem Biophys.

[28] Schmeichel KL, Bissell MJ (2003). Modeling tissue-specific signaling and organ function in three dimensions. J Cell Sci.

[29] Miller RK, Genbacev O, Turner MA, Aplin JD, Caniggia I, Huppertz B (2005). Human placental explants in culture: approaches and assessments. Placenta.

[30] LaMarca HL, Ott CM, Honer Zu Bentrup K, Leblanc CL, Pierson DL, Nelson AB, Scandurro AB, Whitley GS, Nickerson CA, Morris CA (2005). Three-dimensional growth of extravillous cytotrophoblasts promotes differentiation and invasion. Placenta.

[31] Liu H, Collins SF, Suggs LJ (2006). Three-dimensional culture for expansion and differentiation of mouse embryonic stem cells. Biomaterials.

[32] Liu H, Lin J, Roy K (2006). Effect of 3D scaffold and dynamic culture condition on the global gene expression profile of mouse embryonic stem cells. Biomaterials.

[33] Gerami-Naini B, Dovzhenko OV, Durning M, Wegner FH, Thomson JA, Golos TG (2004). Trophoblast differentiation in embryoid bodies derived from human embryonic stem cells. Endocrinology.

[34] Wolfgang MJ, Marshall VS, Eisele SG, Schotzko ML, Thomson JA, Golos TG (2002). Efficient method for expressing transgenes in nonhuman primate embryos using a stable episomal vector. Mol Reprod Dev.

[35] Wolfgang MJ, Eisele SG, Knowles L, Browne MA, Schotzko ML, Golos TG (2001). Pregnancy and live birth from nonsurgical transfer of in vivo- and in vitro-produced blastocysts in the rhesus monkey. J Med Primatol.

[36] Wolfgang MJ, Eisele SG, Browne MA, Schotzko ML, Garthwaite MA, Durning M, Ramezani A, Hawley RG, Thomson JA, Golos TG (2001). Rhesus monkey placental transgene expression after lentiviral gene transfer into preimplantation embryos. Proc Natl Acad Sci U S A.

[37] McKiernan SH, Bavister BD, Tasca RJ (1991). Energy substrate requirements for in-vitro development of hamster 1- and 2-cell embryos to the blastocyst stage. Hum Reprod.

[38] McKiernan SH, Clayton MK, Bavister BD (1995). Analysis of stimulatory and inhibitory amino acids for development of hamster one-cell embryos in vitro. Mol Reprod Dev.

[39] McKiernan SH, Bavister BD (1998). Gonadotrophin stimulation of donor females decreases post-implantation viability of cultured one-cell hamster embryos. Hum Reprod.

[40] Rozner AE, Durning M, Kropp J, Wiepz GJ, Golos TG (2016). Macrophages modulate the growth and differentiation of rhesus monkey embryonic trophoblasts. Am J Reprod Immunol.

[41] Schramm RD, Paprocki AM, VandeVoort CA (2003). Causes of developmental failure of in-vitro matured rhesus monkey oocytes: impairments in embryonic genome activation. Hum Reprod.

[42] McKiernan SH, Bavister BD (2000). Culture of one-cell hamster embryos with water soluble vitamins: pantothenate stimulates blastocyst production. Hum Reprod.

[43] Seshagiri PB, Hearn JP (1993). In-vitro development of in-vivo produced rhesus monkey morulae and blastocysts to hatched, attached, and post-attached blastocyst stages: morphology and early secretion of chorionic gonadotropin. Hum Reprod.

[44] Ziegler TE, Matteri RL, Wegner FH (1993). Detection of urinary gonadotropins in Callitrichid monkeys with a sensitive immunoassay based upon a unique monoclonal-antibody. Am J Primatol.

[45] Munro CJ, Laughlin LS, Illera JC, Dieter J, Hendrickx AG, Lasley BL (1997). ELISA for the measurement of serum and urinary chorionic gonadotropin concentrations in the laboratory macaque. Am J Primatol.

[46] Bielert C, Czaja JA, Eisele S, Scheffler G, Robinson JA, Goy RW (1976). Mating in the rhesus monkey (Macaca mulatta) after conception and its relationship to oestradiol and progesterone levels throughout pregnancy. J Reprod Fertil.

[47] Slukvin II, Lunn DP, Watkins DI, Golos TG (2000). Placental expression of the nonclassical MHC class I molecule Mamu-AG at implantation in the rhesus monkey. Proc Natl Acad Sci U S A.

[48] Bondarenko GI, Burleigh DW, Durning M, Breburda EE, Grendell RL, Golos TG (2007). Passive immunization against the MHC class I molecule Mamu-AG disrupts rhesus placental development and endometrial responses. J Immunol.

[49] McConkey CA, Delorme-Axford E, Nickerson CA, Kim KS, Sadovsky Y, Boyle JP, Coyne CB (2016). A three-dimensional culture system recapitulates placental syncytiotrophoblast development and microbial resistance. Sci Adv.

[50] Enders AC, Meyers S, Vandevoort CA, Douglas GC (2005). Interactions of macaque blastocysts with epithelial cells in vitro. Hum Reprod.

[51] Pankov R, Cukierman E, Katz BZ, Matsumoto K, Lin DC, Lin S, Hahn C, Yamada KM (2000). Integrin dynamics and matrix assembly: tensin-dependent translocation of alpha(5)beta(1) integrins promotes early fibronectin fibrillogenesis. J Cell Biol.

[52] Burrows TD, King A, Smith SK, Loke YW (1995). Human trophoblast adhesion to matrix proteins: inhibition and signal transduction. Hum Reprod.

[53] Davidson LM, Coward K (2016). Molecular mechanisms of membrane interaction at implantation. Birth Defects Res C Embryo Today.

[54] Fisher SJ, Cui TY, Zhang L, Hartman L, Grahl K, Zhang GY, Tarpey J, Damsky CH (1989). Adhesive and degradative properties of human placental cytotrophoblast cells in vitro. J Cell Biol.

[55] Aplin JD, Haigh T, Jones CJ, Church HJ, Vicovac L (1999). Development of cytotrophoblast columns from explanted first-trimester human placental villi: role of fibronectin and integrin alpha5beta1. Biol Reprod.

[56] Blankenship TN, Enders AC (1997). Trophoblast cell-mediated modifications to uterine spiral arteries during early gestation in the macaque. Acta Anat (Basel).

[57] Kliman HJ, Nestler JE, Sermasi E, Sanger JM, Strauss JF (1986). Purification, characterization, and in vitro differentiation of cytotrophoblasts from human term placentae. Endocrinology.

[58] Seshagiri PB, Terasawa E, Hearn JP (1994). The secretion of gonadotrophin-releasing hormone by peri-implantation embryos of the rhesus monkey: comparison with the secretion of chorionic gonadotrophin. Hum Reprod.

[59] Blaschitz A, Weiss U, Dohr G, Desoye G (2000). Antibody reaction patterns in first trimester placenta: implications for trophoblast isolation and purity screening. Placenta.

[60] Haigh T, Chen C, Jones CJ, Aplin JD (1999). Studies of mesenchymal cells from 1st trimester human placenta: expression of cytokeratin outside the trophoblast lineage. Placenta.

[61] Muhlhauser J, Crescimanno C, Kasper M, Zaccheo D, Castellucci M (1995). Differentiation of human trophoblast populations involves alterations in cytokeratin patterns. J Histochem Cytochem.

[62] DaSilva-Arnold S, James JL, Al-Khan A, Zamudio S, Illsley NP (2015). Differentiation of first trimester cytotrophoblast to extravillous trophoblast involves an epithelial-mesenchymal transition. Placenta.

[63] Boyson JE, Iwanaga KK, Golos TG, Watkins DI (1997). Identification of a novel MHC class I gene, Mamu-AG, expressed in the placenta of a primate with an inactivated G locus. J Immunol.

[64] Slukvin II, Boyson JE, Watkins DI, Golos TG (1998). The rhesus monkey analogue of human lymphocyte antigen-G is expressed primarily in villous syncytiotrophoblasts. Biol Reprod.

[65] Ryan AF, Grendell RL, Geraghty DE, Golos TG (2002). A soluble isoform of the rhesus monkey nonclassical MHC class I molecule Mamu-AG is expressed in the placenta and the testis. J Immunol.

