# Supplementary material for: Trophoblast differentiation, invasion and hormone secretion in a three-dimensional in vitro implantation model with rhesus monkey embryos
Source: Reprod Biol Endocrinol. 2018 Mar 16;16:24. doi: 10.1186/s12958-018-0340-3 (PMC5857108; doi:10.1186/s12958-018-0340-3)
Supplement: Supplementary file 1 — Schematic illustration of rhesus macaque embryo culture in the Matrigel-feeder cell 3-D in vitro implantation system. The Matrigel carrier was gelled onto a coverslip then placed into a 35 mm well containing feeder layer of Buffalo Rat Liver cells, followed by injection of one rhesus macaque blastocyst stage embryo into each Matrigel carrier. (PPTX 41 kb) [file 12958_2018_340_MOESM1_ESM.pptx]

## Slide 1
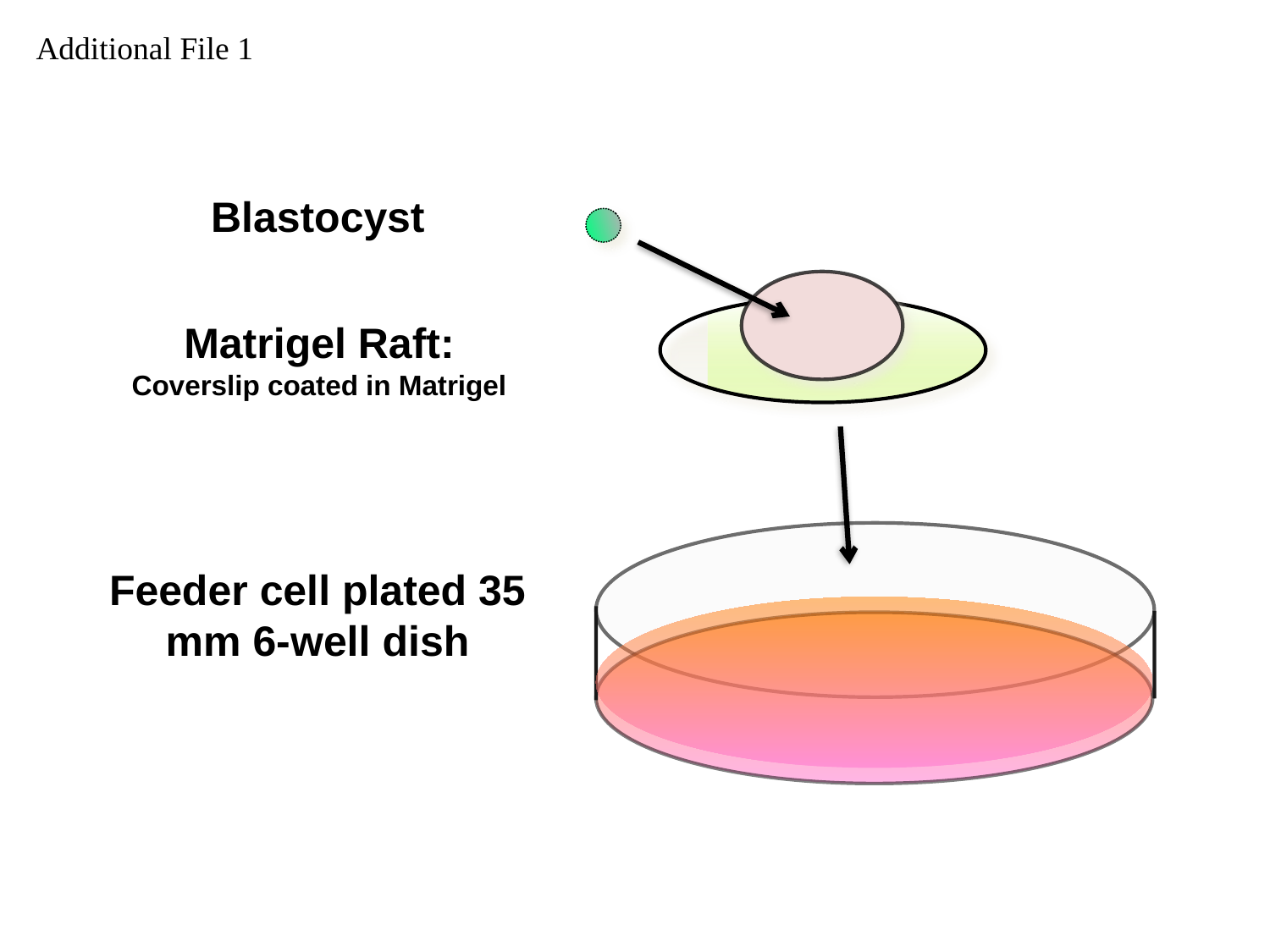

Additional File 1
Blastocyst
Matrigel Raft:
Coverslip coated in Matrigel
Feeder cell plated 35 mm 6-well dish
