# Supplementary material for: Trophoblast differentiation, invasion and hormone secretion in a three-dimensional in vitro implantation model with rhesus monkey embryos
Source: Reprod Biol Endocrinol. 2018 Mar 16;16:24. doi: 10.1186/s12958-018-0340-3 (PMC5857108; doi:10.1186/s12958-018-0340-3)
Supplement: Supplementary file 2 — Antibodies used for immunohistochemical staining on rhesus macaque embryo sections. (DOCX 72 kb) [file 12958_2018_340_MOESM2_ESM.docx]

**Additional File 2.** Antibodies used for immunohistochemical staining on rhesus macaque embryo sections.

| **Antigen** | **Clone** | **Dilution of Ab** | **Working Conc. (μg/mL)** | **Dilution of IgG** | **Major Specificity** | **Supplier** |
| --- | --- | --- | --- | --- | --- | --- |
| CD31 | JC70A  (mouse IgG1, kappa) | 1:80 | 4.3125 | 1:232 | Endothelial cells | Dako |
| CG | 518B7  (mouse IgG1) | 1:800 | 2.5-5 | 1:400 | Trophoblastic epithelium | UC-Davis |
| Cytokeratin | CAM 5.2  (mouse IgG2a) | 1:200 | 0.125 | 1:8800 | Most epithelial cells, with the exception of stratified squamous epithelium | Becton Dickinson |
| MHC  class I | HC10  (mouse IgG2a) | 1:200 | 7.75 | 1:142 | MHC class I | Harlan |
| Ki-67 | MM1  (mouse IgG1) | 1:150 | 0.37 | 1:2703 | Components of the nucleolus during G1, S, G2 and M phases | VECTOR |
| NeuN | MAB377  (mouse IgG1) | 1:8000 | 0.5 | 1:2000 | Most neuronal cell types | Chemicon |
| Vimentin | V9  (mouse IgG1) | 1:4000 | 1.275 | 1:784 | Fibroblasts, endothelial cells, lymphoid tissue, melanocytes | Sigma |
| von Willebrand Factor | F8/86  (mouse IgG1, kappa) | 1:50 | 4.8 | 1:208 | Endothelial cells, megakaryocytes and megacaryoblasts | Dako |
