# Supplementary figures and images for: Trophoblast differentiation, invasion and hormone secretion in a three-dimensional in vitro implantation model with rhesus monkey embryos
Source: Reprod Biol Endocrinol. 2018 Mar 16;16:24. doi: 10.1186/s12958-018-0340-3 (PMC5857108; doi:10.1186/s12958-018-0340-3)

## Slide 1
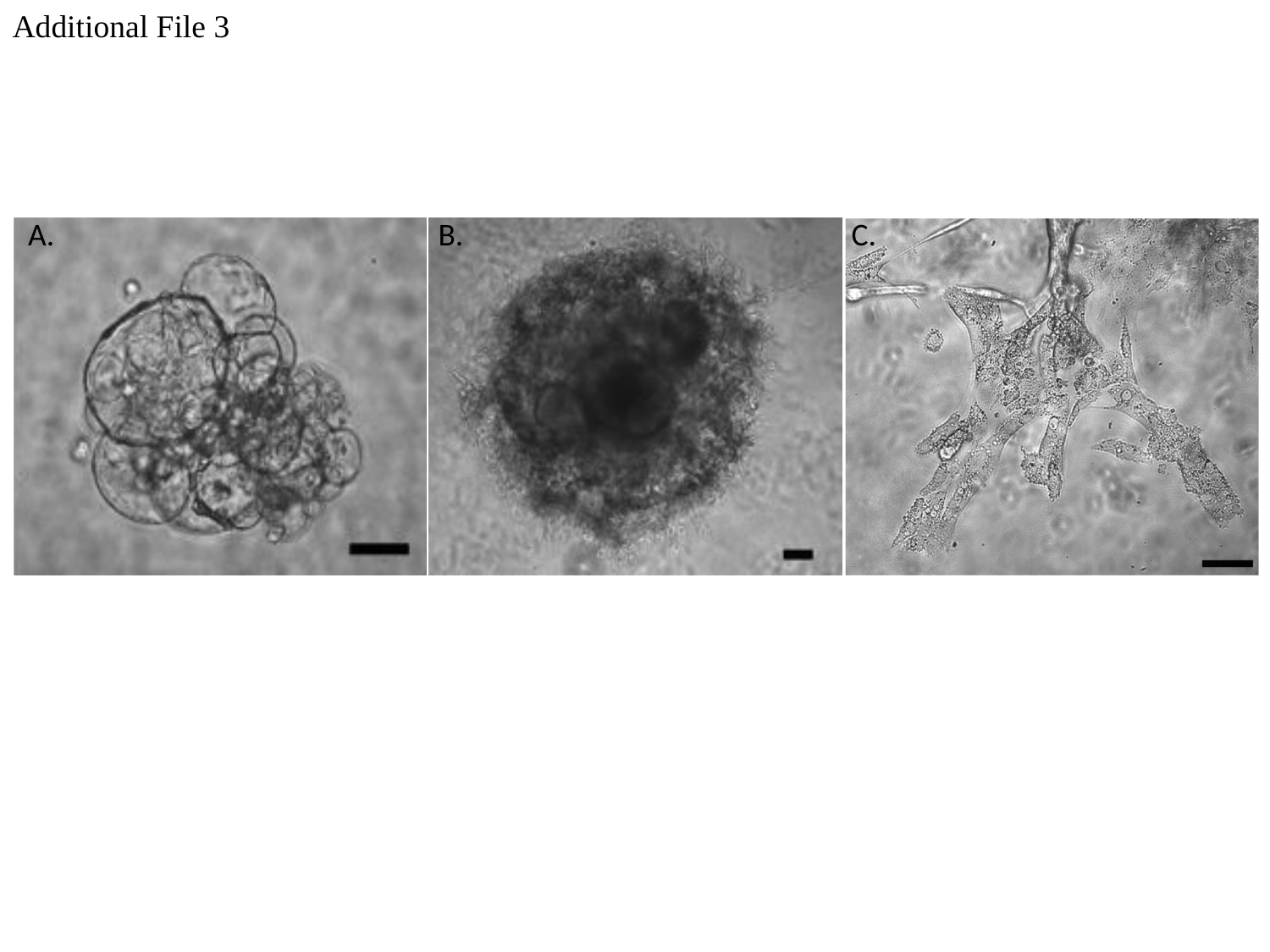

Additional File 3
A.
B.
C.

Supplement: Supplementary file 3 — Branching structure of the trophoblastic protrusion derived from embryo embedded in Matrigel with BRL feeder cell co-culture. Scale bar = 100 μm. (PPTX 715 kb) [file 12958_2018_340_MOESM3_ESM.pptx]

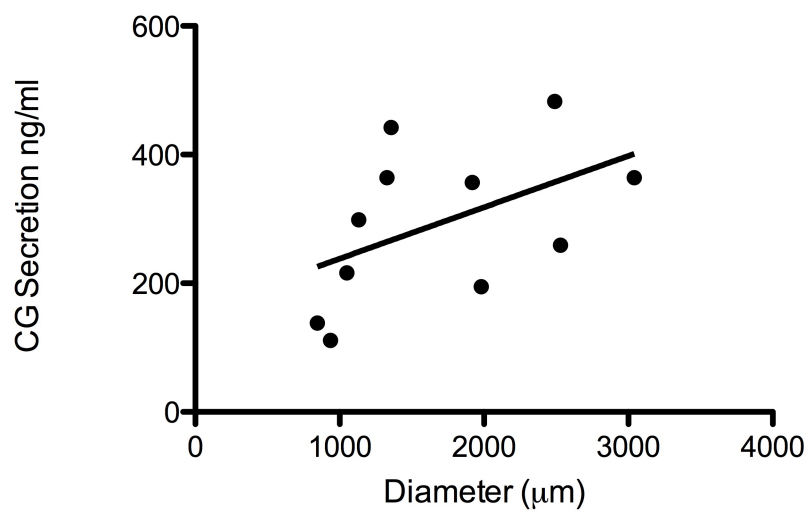

Supplement: Supplementary file 4 — Secretion of CG correlated with embryo size. The peak secretion of CG into culture medium in embryos presented in Fig. 3 is plotted against the maximal growth of embryos through days 18–21 of culture. The line presented was derived by best-fit linear regression analysis. Estimation of the correlation coefficient (Spearman’s nonparametric test) indicted that the correlation was not statistically significant (P = 0.093). (PDF 206 kb) [file 12958_2018_340_MOESM4_ESM.pdf]
